# Associations of Anthropometry and Handgrip Strength with Bioelectrical Impedance Parameters in Irritable Bowel Syndrome: A Cross-Sectional Study

**DOI:** 10.3390/nu18213711

**Published:** 2026-11-09

**Authors:** Jiří Vejmelka, Martina Kollerová, Ivana Pražanová, Khanh Ha Pham, Magdalena Trusinová, Pavel Kohout

**Affiliations:** 1 Department of Internal Medicine and Clinical Nutrition Centre, Third Faculty of Medicine, Thomayer University Hospital, Charles University, Vídeňská 800, 140 59 Prague 4, Czech Republic; 2 Nutrition and Dietetics Centre, Thomayer University Hospital, Vídeňská 800, 140 59 Prague 4, Czech Republic; 3 Department of Hygiene, Epidemiology and Preventive Medicine, Third Faculty of Medicine, Charles University, Ruská 87, 100 00 Prague 10, Czech Republic

**Keywords:** irritable bowel syndrome, body composition, bioelectrical impedance analysis, phase angle, handgrip strength, anthropometry, skinfold thickness, mid-upper arm muscle circumference

## Abstract

Background/Objectives: Simple anthropometry may complement nutritional assessment in irritable bowel syndrome (IBS). We examined the associations of arm anthropometry and handgrip strength (HGS) with bioelectrical impedance analysis (BIA) parameters. Methods: This cross-sectional baseline analysis included 55 adults with IBS-D or IBS-M for valid non-adiposity measurements; 54 contributed valid paired body-fat estimates, and 54 had valid dominant-hand HGS. Results: MUAMC correlated with BIA-derived skeletal muscle mass within men (Spearman’s ρ = 0.748) and women (ρ = 0.673). After adjustment for sex, age, height and body weight, the MUAMC association was attenuated (β = 0.0337 kg/mm, 95% CI 0.0059–0.0616; *p* = 0.0187). MUAMC was correlated with phase angle in the pooled sample (ρ = 0.806), but sex-related differences were exploratory. In a sensitivity model using the impedance index, the association was not significant after further adjustment for body weight (β = 0.0670, 95% CI −0.0087 to 0.1427; *p* = 0.0816). HGS correlated with BIA-derived skeletal muscle mass before adjustment (ρ = 0.769; *n* = 54), whereas an adjusted association was not established. BIA- and skinfold-derived body-fat estimates were correlated but showed proportional bias, limiting interchangeability. Conclusions: MUAMC is associated with BIA-derived muscle-related parameters, but mathematical coupling, body size and the absence of a criterion method limit causal and validation claims. Findings from this exploratory single-centre analysis require independent confirmation.

## 1. Introduction

Irritable bowel syndrome (IBS) is a disorder of gut–brain interaction characterised by recurrent abdominal pain and altered bowel habits. Dietary modification is central to symptom management for many patients, but food avoidance and restrictive eating can reduce energy and nutrient intake [1,2,3]. Nutritional assessment is therefore relevant even in the absence of structural gastrointestinal disease.

Body mass index (BMI) does not distinguish fat from lean tissue or characterise muscle function. Consequently, a normal BMI may provide an incomplete picture of nutritional status. Recommendation 20 of the joint ESPEN/UEG guideline supports nutritional screening in patients with IBS at diagnosis and regularly thereafter (at least annually), including malnutrition, sarcopenia, overweight and obesity (Good Practice Point; 97% expert consensus) [4].

Bioelectrical impedance analysis (BIA) offers a practical, non-invasive approach to estimating body composition. Access to equipment and sensitivity to hydration and measurement conditions can, however, constrain its use. Arm circumference and triceps skinfold thickness can be combined to derive mid-upper arm muscle circumference (MUAMC), while measurements at several skinfold sites can estimate percentage body fat. These portable, inexpensive measures may be useful in outpatient assessment. Handgrip strength (HGS) provides a complementary measure of muscle function.

Evidence linking these bedside measures to BIA parameters in IBS remains limited. Phase angle (PhA) has been assessed in IBS dietary-intervention research [5], but its relationship with simple arm anthropometry in this population is less well characterised. PhA provides an impedance-derived parameter distinct from estimated muscle mass; age, sex and body size must be considered when interpreting its associations [6].

We assessed the relationships of skinfold-derived percentage body fat, MUAMC and HGS with BIA-derived body-composition measures in adults with diarrhoea-predominant (IBS-D) or mixed IBS (IBS-M). The principal comparisons concerned body-fat estimates from the two methods and the associations of MUAMC and HGS with BIA-derived muscle mass. The association between MUAMC and PhA was examined as a secondary exploratory analysis.

## 2. Materials and Methods

### 2.1. Study Design and Participants

This ancillary cross-sectional analysis used pre-intervention baseline data from MISCEAT, a randomised, double-blind, placebo-controlled crossover study of faecal microbiota transplantation in IBS (ClinicalTrials.gov NCT04899869) [7]. Participants were enrolled at the Department of Internal Medicine, Thomayer University Hospital, Prague, Czech Republic, between July 2021 and September 2023. Of the 61 participants randomised in the parent study, 55 had both baseline BIA and anthropometric measurements available and were initially eligible for this ancillary cross-sectional analysis. Because the present analysis used only pre-intervention measurements, eligibility did not require receipt of the allocated intervention or completion of follow-up. One participant had physiologically invalid zero body-fat estimates; these were excluded only from adiposity analyses (n = 54), while independently verified valid non-adiposity measurements were retained (n = 55). No a priori sample-size calculation was performed specifically for this ancillary cross-sectional analysis; its sample size was determined by the availability of baseline BIA and anthropometric data. The parent trial was not powered for the present sex-stratified associations or interaction analyses. Participants were adults aged 18–65 years with IBS-D or IBS-M according to Rome IV criteria; the parent study eligibility criteria are summarised in Table 1.

### 2.2. Bioelectrical Impedance Analysis

Body composition was assessed using the seca mBCA 515 Medical Body Composition Analyzer (seca, Hamburg, Germany), with seca analytics 115 software (version 1.4.552.5749). The device measures body weight and estimates fat mass, fat-free mass, total body water and skeletal muscle mass using model-based BIA algorithms. Skeletal muscle mass is not measured directly, and its prediction may share body-size determinants with anthropometric measures. It uses eight hand and foot electrodes and multiple frequencies spanning 1–1000 kHz [8]. Phase angle (φ; 50 kHz) was taken from the recorded BIA output and analysed separately from the model-derived body-composition estimates. BIA assessment excluded individuals with an implanted pacemaker or defibrillator, an active prosthesis, or a connection to electronic medical equipment. Pregnancy was excluded by the study protocol; cardiac arrhythmia required consultation with the treating physician.

The published development model for total skeletal muscle mass in the seca mBCA 515/525 system [8] uses height squared divided by whole-body resistance at 50 kHz (height^2^/R50), reactance at 50 kHz (Xc50), body weight, sex, and segmental resistance/reactance indices. Its complete regression coefficients and intercept were not publicly disclosed for commercial reasons. The published model describes the relevant device family, but identity with the exact installed software implementation has not been independently confirmed. Whole-body R50 and Xc50 were recovered from the original seca mBCA 515 assessments. The impedance index was calculated as height (cm) squared divided by R50 (Ω).

Participants received written preparation instructions: no food or drink except small amounts of water, no alcohol or caffeinated beverages, and limited physical activity for at least 8 h before assessment. They were instructed to empty their bladder immediately before measurement.

Measurements were performed in underwear, with a light T-shirt permitted, after breathing had returned to normal and any perspiration had dried. Participants stood barefoot on the foot electrodes and held the hand electrodes at a height-appropriate grip position, with the arms approximately 45° from the trunk and no contact between the limbs. They remained still and did not speak during measurement. Mobile phones were kept at least 1 m from the device. BIA, anthropometry and HGS were assessed on the same day. All participants underwent clinical examination by a physician, and none exhibited clinically apparent signs of dehydration. Menstrual-cycle phase was not standardised or systematically recorded.

### 2.3. Anthropometry and Skinfold Measurements

BMI was calculated as body weight (kg) divided by squared height (m^2^).

*BMI* (kg/m^2^) = *body weight* (kg)/[*height* (m)]^2^

Skinfold-derived body-fat percentage was estimated from age- and sex-specific Durnin–Womersley conversion tables [9] using the sum of four skinfolds (biceps, triceps, subscapular and suprailiac). A Harpenden calliper was used on the right side of the body, irrespective of handedness. Each skinfold was measured three times, and the median of the three readings at each site was used to calculate the sum. The corresponding age- and sex-specific tabulated body-fat percentage was recorded. The tabular approach was used in the original assessment; individual body-fat percentages were not calculated directly from regression equations.

*S* = *biceps* + *triceps* + *subscapular* + *suprailiac skinfold thickness*

*D* = *a* − *b* × *log*_10_(*S*)

The Durnin–Womersley tables are based on age- and sex-specific relationships between the base-10 logarithm of the sum of four skinfolds and body density, with subsequent conversion to body-fat percentage using the Siri equation [10]. The original assessments used the tabulated values rather than direct calculation from the equations.

*Body fat* (%) = 495 / *D* − 450

Mid-upper arm circumference (MUAC) was measured once on each arm using a non-stretchable tape at the midpoint between the acromion and olecranon, with the arm relaxed alongside the body and without compressing the soft tissues. Mid-upper arm muscle circumference (MUAMC) was derived from the right-arm MUAC and the median of three right triceps skinfold thickness (TSF) measurements. Both measures were expressed in millimetres. MUAMC was calculated as MUAC − π × TSF, using full numerical precision of π.

*MUAMC* (mm) = *MUAC* (mm) − *π* × *TSF* (mm)

Waist circumference was measured at the level of the umbilicus by clinical dietitians following a common anthropometric protocol. Formal inter-rater reliability testing was not performed, and the assessors were not blinded to the BIA results.

### 2.4. Handgrip Strength

Handgrip strength (HGS) was assessed as an integral component of routine clinical nutritional evaluation during the baseline study visit using a Saehan SH5008 bulb-type dynamometer. The compressible bulb was positioned in the palm and squeezed using the whole hand, including the thumb; the device has no adjustable handle. Participants were seated with the shoulder in neutral rotation, the elbow flexed at approximately 90°, and the forearm in a neutral position. Following instructions, one maximal squeeze was measured on the right hand first and then on the left, without a preceding familiarisation trial. Dominance was identified from self-reported handedness (right- or left-handed), not from the higher grip reading. Measurements were recorded on the inner pounds-force scale and converted to kilogram-force (1 lbf = 0.45359237 kgf), conventionally reported as kg in clinical tables and figures. The dominant-hand value was used for the primary analysis. One participant could not undergo dominant-hand measurement because of a hand orthosis; this value was treated as missing. The bulb device is not interchangeable with Jamar-type dynamometers for clinical reference thresholds.

### 2.5. Clinical and Symptom Assessment

IBS symptom severity was assessed at baseline using the IBS Severity Scoring System (IBS-SSS; range 0–500, with higher scores indicating greater symptom severity). The baseline questionnaire also recorded the average daily number of diarrhoeal stools and predominant Bristol stool form over the preceding seven days.

### 2.6. Statistical Analysis

Continuous variables were summarised as mean ± standard deviation and categorical variables as n (%). Associations were assessed using Spearman rank correlations with paired percentile-bootstrap 95% confidence intervals (1800 resamples; fixed random seed 42) and corresponding two-sided *p* values. Analyses used available paired observations without imputation. Skinfold-derived body-fat values were taken from the study database using the original age- and sex-specific Durnin–Womersley tables. One participant’s non-physiological zero body-fat values were excluded from adiposity analyses only; other measurements were retained for sensitivity analysis, without assuming independent validity of all BIA-derived outputs. The agreement between BIA-derived and skinfold-derived percentage body fat was assessed using Bland–Altman methods [11]. Differences were defined as BIA minus skinfold values. Overall descriptive limits of agreement were calculated as the mean difference ± 1.96 SD of differences. Proportional bias was assessed by ordinary least-squares regression of differences on paired means; regression-based conditional limits were considered where appropriate. No a priori clinically acceptable difference between the methods had been specified. Diagnostics of the regression-based approach require consideration of residual variation, rather than relying solely on the distribution of unadjusted differences. Regression-based conditional limits of agreement were calculated as

$${\mathrm{LoA}}_{\mathrm{conditional}}\left(\bar{m}\right)=\left(a+b\bar{m}\right)\pm 1.96\;s_{\mathrm{res}},$$

where a denotes the intercept, b the regression slope, $\bar{m}$ the paired mean, and s_res_ the residual standard deviation (2.906 percentage points); approximately constant residual variability was assumed.

Sex-stratified Spearman correlations with 1800-resample percentile-bootstrap 95% confidence intervals were also calculated. Within-sex exploratory models of SMM and phase angle on MUAMC were adjusted for age and height with HC3 inference. Separate ordinary least-squares models assessed MUAMC and HGS in relation to BIA-derived muscle mass and MUAMC in relation to PhA, each adjusted for sex, age and height. Standardised coefficients were obtained by scaling the predictor and outcome by their sample standard deviations. An exploratory PhA model added a MUAMC-by-sex interaction, with MUAMC centred at its overall mean. A separate SMM model additionally adjusted for body weight. All covariate-adjusted models used HC3 heteroscedasticity-consistent standard errors for 95% confidence intervals and two-sided *p* values, with a Student t reference distribution and residual degrees of freedom. HC3 was applied consistently to allow for potentially unequal residual variances. Leave-one-out analyses refitted the interaction model after omitting each participant, assessing changes in the estimated coefficients using conventional OLS refits. The leave-one-out output was used as a sensitivity check and was not interpreted as HC3-robust significance testing.

Additional exploratory analyses examined MUAMC in relation to the impedance index (height^2^/R50) using HC3-robust regression adjusted for sex and age, then additionally for height, and finally for body weight. Sensitivity analyses compared the 55-person cohort with the original 54-person cohort. Zero body-fat estimates were treated as invalid for adiposity analyses only; other available measurements from this participant were retained for sensitivity analysis, acknowledging uncertainty in the validity of model-derived SMM for applicable analyses. These secondary analyses were not corrected for multiple comparisons.

Tests were two-sided, with a nominal significance level of α = 0.05. Effect estimates and confidence intervals were interpreted alongside *p* values. No adjustment for multiple testing was applied; the secondary MUAMC–PhA analysis and sex interaction were considered exploratory. Calculations were performed in Python 3.12.14 using NumPy 2.3.5, SciPy 1.17.0 and pandas 2.2.3.

## 3. Results

### 3.1. Participant Characteristics

The revised analytical cohort comprised 55 adults with IBS-D or IBS-M for available valid non-adiposity measurements. The participant with non-physiological zero body-fat outputs was retained for valid non-adiposity analyses but excluded from adiposity comparisons (n = 54). Dominant-hand HGS was available in 54 participants. Participant characteristics are presented in Table 2.

### 3.2. Adiposity: BIA and Skinfold Measurements

**Figure 1:**
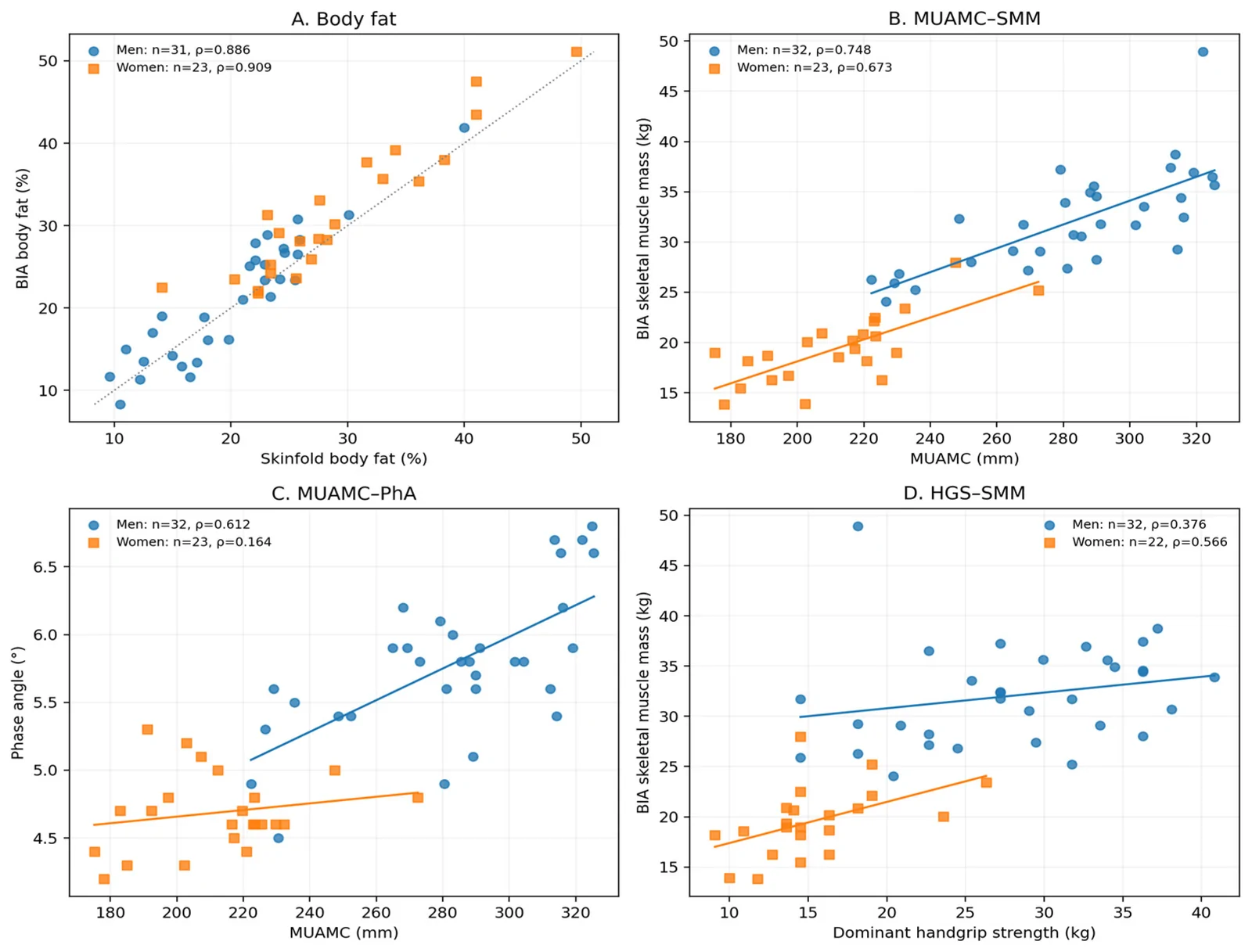
Associations between bedside measures and BIA-derived parameters: (**A**) Paired percentage body-fat estimates (*n* = 54). (**B**) MUAMC and BIA-derived skeletal muscle mass (*n* = 55). (**C**) MUAMC and phase angle (*n* = 55). (**D**) Dominant-hand HGS, reported in kg, and BIA-derived skeletal muscle mass (*n* = 54).

**Figure 2:**
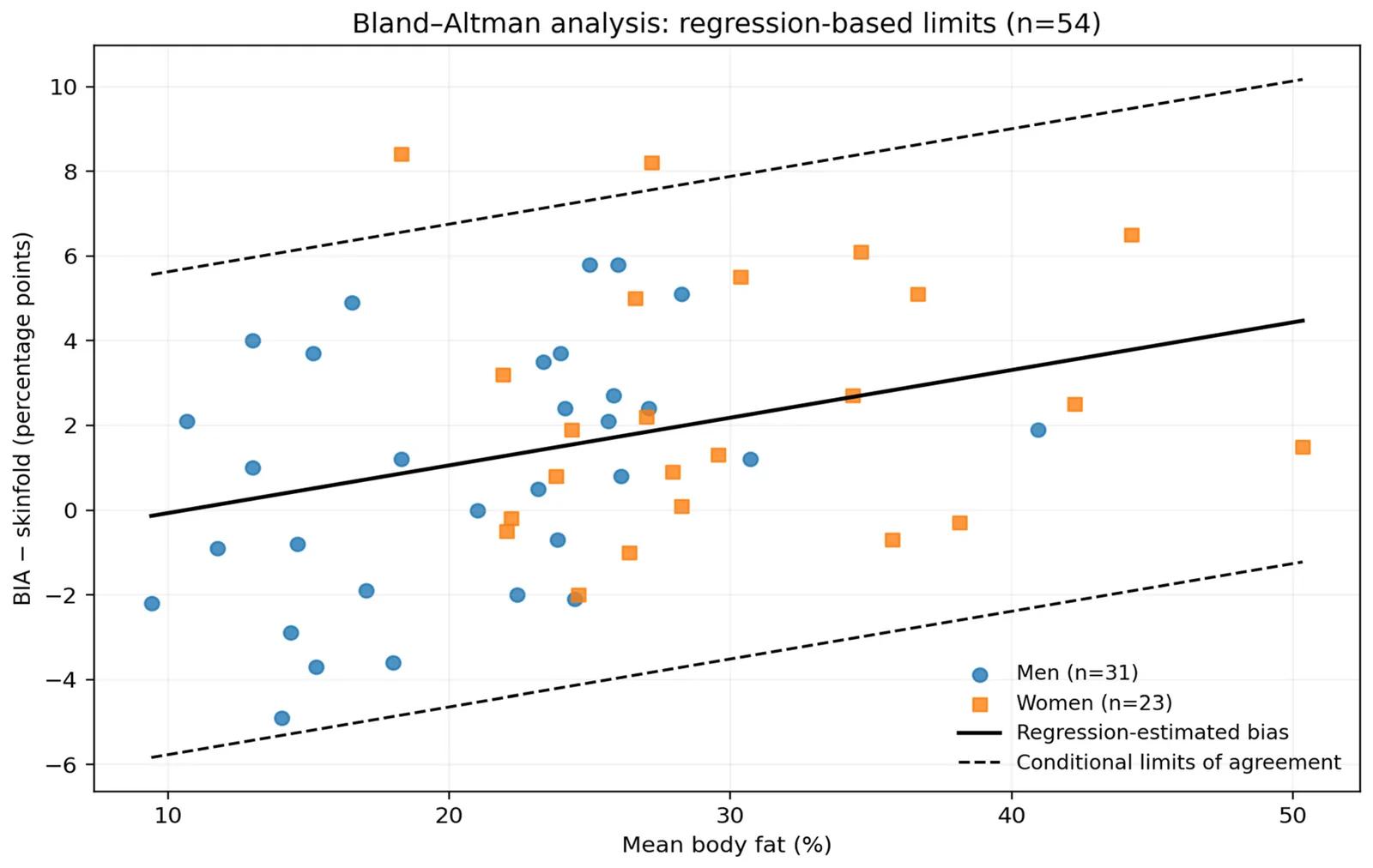
Bland–Altman comparison of BIA-derived and skinfold-derived percentage body fat (*n* = 54). Differences are BIA minus skinfold body fat in percentage points. The solid line indicates regression-estimated proportional bias; dashed lines show conditional limits of agreement calculated as fitted bias ± 1.96 × residual SD. The overall residual SD was 2.906 percentage points. The limits assume approximately constant residual variability and do not establish clinical interchangeability.

Among 54 participants with valid paired body-fat estimates, BIA and skinfold percentage body fat were positively correlated (Spearman ρ = 0.913). The mean BIA-minus-skinfold difference was 1.60 percentage points, with overall descriptive 95% limits of agreement from −4.37 to 7.56 percentage points. Regression-based Bland–Altman analysis indicated proportional bias (slope = 0.113; *p* = 0.017). Regression-based conditional limits were calculated as (a + b × paired mean) ± 1.96 × residual SD, where a is the intercept, b the regression slope, and the residual SD was 2.906 percentage points. Sex-specific slopes were 0.157 in men and 0.026 in women, with mean biases of 0.939 and 2.487 percentage points, respectively. Regression parameters are provided in Supplementary Table S6. Without a criterion method or a prespecified clinically acceptable difference, interchangeability cannot be established. The comparison is illustrated in Figure 1A and the agreement analysis in Figure 2.

### 3.3. Muscle-Related Measures

In the revised non-adiposity cohort (*n* = 55), MUAMC was associated with BIA-derived skeletal muscle mass (Spearman ρ = 0.913). Dominant-hand HGS was associated with BIA-derived muscle mass (ρ = 0.769; *n* = 54). In an HC3-robust model adjusted for sex, age and height, MUAMC was associated with muscle mass (unstandardised β = 0.1051 kg/mm, *p* < 0.001). Additional adjustment for body weight attenuated the association (β = 0.0337 kg/mm, 95% CI 0.0059 to 0.0616; *p* = 0.0187). These estimates do not establish independent criterion validity. These associations are illustrated in Figure 1B,D. The adjusted skeletal-muscle-mass regression coefficients are detailed in Supplementary Table S1.

Within-sex analyses showed smaller associations than the pooled analysis, consistent with a contribution from between-sex differences. In the revised cohort, the HGS association was not statistically significant after adjustment for sex, age and height (HC3 *p* = 0.3914). This attenuation does not demonstrate physiological independence of strength and muscle quantity. Within-sex correlation estimates with bootstrap 95% confidence intervals and adjusted models are presented in Supplementary Tables S9 and S10; multicollinearity diagnostics are provided in S3–S4. In VIF diagnostics, MUAMC VIF was approximately 3.06 in the basic model and 5.02 after adding body weight (*n* = 55), indicating increased collinearity in the latter model. Supplementary Table S7 provides the dominant- and non-dominant-hand HGS models; Supplementary Table S9 provides within-sex rank correlations, and Supplementary Table S10 reports the corresponding adjusted within-sex MUAMC models.

Sensitivity analysis comparing the 54- and 55-participant non-adiposity datasets yielded similar MUAMC–muscle mass correlations (ρ = 0.909 and 0.913, respectively). The observation with the highest Cook’s distance in the weight-adjusted SMM model was distinct from the participant with invalid body-fat estimates. Excluding this influential observation yielded a positive MUAMC coefficient (β = 0.0436 kg/mm; 95% HC3 CI 0.0241 to 0.0632; *p* < 0.001; *n* = 54). Detailed coefficients, 95% confidence intervals, *p* values and VIF diagnostics are reported in the Supplementary Materials. Model diagnostics suggested possible misspecification in the weight-adjusted SMM model (RESET *p* = 0.0014), and its maximum Cook’s distance was 1.924. These findings warrant cautious interpretation of the model. The sample-size sensitivity comparison is detailed in Supplementary Table S2, while regression diagnostics are reported in Supplementary Table S4. Additional rank-correlation and sample-sensitivity results are provided in Supplementary Table S8.

### 3.4. MUAMC and Phase Angle

MUAMC correlated with phase angle in the revised non-adiposity cohort (Spearman ρ = 0.806; *n* = 55). The HC3-robust regression adjusted for sex, age and height yielded an unstandardised MUAMC coefficient of 0.0107 degrees/mm (*p* < 0.001). Sex-specific and expanded interaction analyses are presented as exploratory because of limited subgroup size, restricted phase-angle variability among women and sensitivity to individual observations. The association is illustrated in Figure 1C.

The exploratory sex-by-MUAMC interaction was not treated as evidence of a confirmed sex-specific biological effect. Centred interaction-model estimates are presented in the updated Supplementary Table S3, and the summary of conventional-OLS leave-one-out coefficient variability is provided in Table S4. Neither table provides leave-one-out HC3 *p* values. Centring MUAMC left the interaction coefficient unchanged (β = 0.0090 degrees/mm; HC3 *p* = 0.0095) and reduced its VIF from 130.60 to 4.96. The interaction and phase-angle regression estimates are presented in Supplementary Table S3.

In an additional sensitivity analysis, MUAMC correlated with the raw impedance index (height^2^/R50; Spearman’s ρ = 0.895; n = 55). In the HC3 model additionally adjusted for sex, age, height and body weight, the MUAMC coefficient was 0.0670 (95% CI −0.0087 to 0.1427; *p* = 0.0816), not reaching conventional statistical significance. The corresponding weight-adjusted SMM model yielded β = 0.0337 kg/mm (95% CI 0.0059 to 0.0616; *p* = 0.0187). In the corresponding n = 54 sensitivity analysis, the weight-adjusted impedance-index association was β = 0.0817 (95% CI 0.0180 to 0.1455; *p* = 0.0131), indicating sensitivity to the analytical sample. These associations remain subject to shared body-size determinants. Model diagnostics suggested possible nonlinearity in the MUAMC–PhA model (quadratic term *p* = 0.0276). This exploratory diagnostic finding should be interpreted cautiously. The impedance-index regression specifications and coefficients are provided in Supplementary Table S5.

In exploratory analyses, the mean daily number of diarrhoeal stools during the preceding seven days was not significantly associated with phase angle (Spearman ρ = −0.108; *p* = 0.431). No clinical signs of dehydration were observed on baseline physician examination, but subclinical fluid shifts were not objectively excluded.

The main bivariate associations between anthropometric, functional, and bioelectrical impedance parameters are summarised in Table 3.

## 4. Discussion

In adults with IBS-D or IBS-M, MUAMC was associated with BIA-derived muscle mass and phase angle, with attenuation after adjustment for body size. Skinfold-derived and BIA-derived body-fat estimates correlated strongly, although individual-level agreement was limited. HGS correlated with BIA-derived muscle mass in the pooled unadjusted analysis, but the adjusted association was not established. These measures represent related, partly overlapping dimensions of nutritional assessment rather than demonstrably independent measures of muscle quantity or function.

The clinical rationale for accessible nutritional assessment in IBS is supported by evidence linking food avoidance and restriction to lower nutrient intake and greater symptom burden [1]. Guidance emphasises dietitian involvement and caution with restrictive diets in patients at risk of malnutrition [2], while emerging evidence on avoidant/restrictive eating among patients with disorders of gut–brain interaction highlights the importance of assessing eating patterns [3]. A dietitian-led 12-week low-FODMAP intervention did not worsen measured nutritional status or nutrient intake, but several nutrient intakes remained below recommendations [12]. These findings support regular nutritional screening and targeted assessment, consistent with the ESPEN/UEG guideline [4].

MUAMC was associated with BIA-derived muscle mass within both sexes as well as in the adjusted analysis. Because it is derived from arm circumference and triceps skinfold thickness, MUAMC is accessible where BIA is unavailable or repeated measurements are needed. Its interpretation nevertheless depends on standardised landmarking and measurement technique: it estimates the muscular component of the upper arm, rather than directly measuring whole-body muscle mass.

The MUAMC–PhA association provides a further link between simple arm anthropometry and bioimpedance assessment. In 135 patients with chronic hepatitis C virus infection, Dorna et al. reported an association between mid-arm muscle circumference and PhA after adjustment for age and sex (*p* = 0.003) [13]. Peres et al. also found that PhA correlated with mid-arm muscle circumference in 66 patients with chronic liver disease [14]. Recent data from patients undergoing post-stroke rehabilitation also linked limb circumference, particularly calf circumference, with whole-body and segmental PhA, although associations involving uncorrected mid-upper arm circumference were comparatively weak [15]. The stronger association observed for MUAMC in our cohort may partly relate to adjustment for triceps skinfold thickness, which is intended to better isolate the muscular component of the arm; however, this interpretation requires direct comparative validation. Our findings extend these observations to an IBS cohort and support further evaluation of arm anthropometry as a complementary component of nutritional and body-composition assessment.

Leave-one-out analyses suggested relative stability of the estimated interaction coefficient, although the statistical evidence for a sex-specific difference remains uncertain. Given the modest sample size, restricted phase-angle variability among women, multicollinearity and the exploratory nature of the analysis, this finding requires independent confirmation.

The body-fat comparison illustrates why correlation alone is insufficient to assess agreement. Despite a strong rank correlation, the overall 95% limits of agreement spanned 11.93 percentage points (−4.37 to 7.56). The difference between methods also increased with mean percentage body fat. The overall descriptive limits are therefore approximations and should not be interpreted as level-specific limits of agreement [11]. The absence of a statistically significant departure from normality does not resolve this proportional bias. No universally applicable threshold can be assumed for individual-level acceptability across these methods; these findings limit substitution of one estimate for the other when assessing an individual patient. Neither BIA nor skinfold estimates were compared with a criterion method, so the observed discrepancy cannot identify which estimate is more accurate.

One participant had zero body-fat estimates from both the seca BIA device and the recorded skinfold table. The four-skinfold sum was 13 mm, below the relevant Durnin–Womersley tabular range. The cause of the device-reported zero BIA fat estimate could not be determined retrospectively. Recorded R50 and Xc50 were internally consistent with the reported phase angle, but this does not independently establish the validity of the proprietary SMM estimate. We therefore excluded the invalid fat estimates from adiposity analyses and examined sensitivity to retaining this participant in non-adiposity models. The participant was distinct from the most influential observation in the weight-adjusted SMM model (Cook’s D = 1.924).

HGS is a measure of muscle strength, but the absence of a significant adjusted association does not demonstrate independence from skeletal muscle quantity. Studies in inflammatory bowel disease have associated HGS with nutritional and body-composition measures and described impaired strength in patients otherwise classified as well nourished [16,17]. The ECCO consensus similarly supports assessment beyond BMI, including body composition and muscle function [18]. Population studies show that absolute HGS varies with sex, age and body size [19,20,21]. In our cohort, adjustment attenuated its relationship with muscle mass, without establishing whether HGS adds independent information beyond muscle quantity. Whether repeated HGS measurements help monitor nutritional change in IBS remains a question for longitudinal research. No familiarisation trial was undertaken; the right hand was always tested first, and calibration documentation for the bulb dynamometer was not available. The bulb dynamometer readings should not be interpreted against reference thresholds developed using hydraulic Jamar-type instruments. Formal inter-rater reliability testing for anthropometry was not conducted. Evidence on the reliability of single versus repeated grip trials is discussed by Coldham et al. [22].

The simultaneous assessment of BIA, detailed anthropometry and HGS is a strength of this study. Limitations include the cross-sectional design, modest sample size, recruitment at one centre and restriction to an IBS-D/M cohort. BIA-derived estimates depend on proprietary prediction algorithms and measurement conditions; no criterion method such as MRI or DXA was available. Anthropometry is operator-dependent; formal inter-rater reliability was not assessed. Although each skinfold was measured three times and its median used, MUAC was measured once on each arm. HGS was measured only once per hand without familiarisation, and calibration documentation was not available for this analysis; bulb-dynamometer readings should not be directly compared with Jamar-based reference norms. The exploratory PhA and interaction analyses were not corrected for multiple comparisons. The restricted phase-angle range in women and small subgroup sizes limit inference on sex interactions. The geometric assumptions of MUAMC may introduce error. Standardised BIA preparation and absence of clinically apparent dehydration do not exclude subclinical hydration variation; objective hydration assessment and menstrual-cycle standardisation were unavailable. Assessors were not blinded to BIA results. Regression diagnostics also identified model-specific concerns about functional form and influential observations, particularly in the weight-adjusted SMM model.

The additional impedance-index analysis reduces dependence on the proprietary seca muscle-mass prediction equation but does not remove shared dependence on body size or hydration. In particular, after further adjustment for body weight, the MUAMC–impedance-index association was attenuated and was not nominally significant in the 55-person cohort (*p* = 0.082). This result should be interpreted alongside the positive association in less-adjusted models and the potential for multicollinearity. The available clinical examination did not identify overt dehydration, but subclinical differences in hydration cannot be excluded. Baseline diarrhoeal activity covered the preceding seven days and is not a direct hydration measurement.

These findings support prospective evaluation of an accessible assessment set combining body weight and BMI, standardised arm anthropometry, skinfolds and HGS. Such studies should establish measurement reproducibility, responsiveness to change and the ability to identify clinically relevant nutritional impairment. The present results provide evidence of association to inform that work.

## 5. Conclusions

In adults with IBS-D or IBS-M, MUAMC was associated with BIA-derived skeletal muscle mass and phase angle after adjustment for sex, age and height. The MUAMC–SMM coefficient was substantially attenuated after additional adjustment for body weight, while the weight-adjusted impedance-index association was sensitive to the inclusion of one participant and was not statistically significant in the full sample. BIA- and skinfold-derived body-fat estimates correlated but showed proportional bias and insufficient evidence for interchangeability. HGS correlated with SMM in unadjusted analyses, but its independent contribution was not established. These exploratory findings require independent confirmation.

## Figures and Tables

**Table 1:** Eligibility criteria for participants in the MISCEAT parent study.

Criterion	Definition
Inclusion	Adults aged 18–65 years
Inclusion	IBS-D or IBS-M diagnosed according to Rome IV criteria
Inclusion	Expected adherence to the study protocol
Inclusion	Written informed consent
Exclusion	Use of antibiotics or probiotics within 1 month before enrolment
Exclusion	History of inflammatory bowel disease, gastrointestinal malignancy, or systemic autoimmune disease
Exclusion	Previous abdominal surgery other than appendectomy, cholecystectomy, hernioplasty, or caesarean section
Exclusion	HIV infection or other active infection
Exclusion	Renal or hepatic disease defined by biochemical workup
Exclusion	Diabetes mellitus or abnormal thyroid function not controlled by thyroid medication
Exclusion	Bipolar disorder or schizophrenia; moderately severe depression (PHQ-9 > 15); anxiety (GAD-7 > 10); or an organic cause explaining IBS symptoms
Exclusion	Current pregnancy or lactation

Abbreviations: GAD-7, Generalised Anxiety Disorder-7; IBS-D, diarrhoea-predominant irritable bowel syndrome; IBS-M, mixed irritable bowel syndrome; PHQ-9, Patient Health Questionnaire-9. Criteria summarised from the published MISCEAT protocol [7].

**Table 2:** Participant characteristics overall and by sex.

Characteristic, Unit	Overall (*n* = 55)	Men (*n* = 32)	Women (*n* = 23)
Age, years	35.3 ± 10.5	35.4 ± 9.2	35.2 ± 12.3
IBS-D; IBS-M, n	48; 7	28; 4	20; 3
IBS-SSS	291.4 ± 80.4	273.9 ± 82.4	315.7 ± 72.4
BMI, kg/m^2^	23.8 ± 5.8	24.6 ± 5.7	22.7 ± 5.8
Waist circumference, cm	84.1 ± 15.2	88.9 ± 14.4	77.3 ± 14.0
Phase angle (50 kHz), degrees	5.3 ± 0.7	5.8 ± 0.5	4.7 ± 0.3
BIA percentage body fat, %	25.6 ± 9.4 (n = 54)	21.2 ± 7.6 (n = 31)	31.5 ± 8.3
BIA muscle mass, kg	26.8 ± 7.7	32.1 ± 5.1	19.4 ± 3.4
MUAMC, mm	253.2 ± 44.8	282.7 ± 31.2	212.1 ± 23.1
Dominant-hand HGS, kg	22.9 ± 8.8 (n = 54)	28.1 ± 7.3	15.3 ± 4.1 (n = 22)

**Table 3:** Associations between anthropometric, functional and BIA measures.

Relationship	*n*	Spearman ρ	95% Bootstrap CI	*p* Value
BIA body fat vs. skinfold body fat	54	0.913	0.831 to 0.953	<0.001
BIA muscle mass vs. MUAMC	55	0.913	0.847 to 0.943	<0.001
BIA muscle mass vs. dominant HGS	54	0.769	0.620 to 0.864	<0.001
MUAMC vs. dominant HGS	54	0.734	0.559 to 0.833	<0.001
MUAMC vs. phase angle	55	0.806	0.690 to 0.880	<0.001

BIA, bioelectrical impedance analysis; CI, confidence interval; HGS, handgrip strength; MUAMC, mid-upper arm muscle circumference; PhA, phase angle (50 kHz). Confidence intervals are percentile bootstrap estimates based on 1800 resamples (seed 42). The MUAMC–PhA comparison is a secondary exploratory analysis.

## Data Availability

The participant-level data are not publicly available because the informed-consent process did not include public sharing of individual-level data. Requests for access may be directed to the corresponding author and will be considered subject to applicable ethical, institutional and data-protection requirements.

## Supplementary Materials

The following supporting information can be downloaded at: https://www.mdpi.com/article/10.3390/nu18213711/s1, Supplementary Tables S1–S10 and the influence-sensitivity summary are provided in the accompanying Supplementary Statistical Analyses. Source code and reproducibility instructions are provided in a separate supplementary code archive. Individual-level participant data are not publicly shared because of ethical and data-protection restrictions. Supplementary table titles: Table S1. Adjusted skeletal muscle mass models; Table S2. Sensitivity to retaining the participant with invalid adiposity values; Table S3. Phase-angle models and exploratory sex interaction; Table S4. Model diagnostics; Table S5. Impedance-index regression models; Table S6. Bland–Altman regression summary; Table S7. Dominant and non-dominant HGS models; Table S8. Rank correlations and sample sensitivity; Table S9. Within-sex Spearman correlations; Table S10. Within-sex, age- and height-adjusted MUAMC models, with additional weight-adjusted SMM models.
