# Supplementary material for: Associations of Anthropometry and Handgrip Strength with Bioelectrical Impedance Parameters in Irritable Bowel Syndrome: A Cross-Sectional Study
Source: Nutrients. 2026 Nov 9;18(21):3711. doi: 10.3390/nu18213711 (PMC13765782; doi:10.3390/nu18213711)
Supplement: Supplementary file 1 [file nutrients-18-03711-s001.zip › nutrients-4620032-supplementary.pdf]

Supplementary Statistical Analyses

MISCEAT anthropometry ancillary analysis | Supplementary Statistical Analyses

These tables report adjusted models, sensitivity analyses and diagnostics reproduced from the accompanying analytical scripts. The findings are observational; interaction and additional sensitivity analyses are exploratory.

Table S1. Adjusted skeletal muscle mass models

Model	Term	β	95% CI	p	VIF
SMM basic	MUAMC	0.1051	0.0638 to 0.1465	<0.001	3.06
SMM basic	Sex	1.8380	0.1025 to 3.5734	0.0384	3.35
SMM basic	Age	-0.0178	-0.0802 to 0.0445	0.5688	1.16
SMM basic	Height	0.2353	0.0822 to 0.3884	0.0033	2.63
SMM + weight	MUAMC	0.0337	0.0059 to 0.0616	0.0187	5.02
SMM + weight	Sex	4.7775	2.7141 to 6.8408	<0.001	3.75
SMM + weight	Age	-0.0683	-0.1206 to -0.0160	0.0115	1.22
SMM + weight	Height	0.1590	0.0584 to 0.2595	0.0026	2.73
SMM + weight	Weight	0.1803	0.0899 to 0.2707	<0.001	2.50

HC3 robust inference; n=55. Higher VIF in the weight-adjusted model warrants cautious interpretation.

Sample-size sensitivity is reported in Table S2. The weight-adjusted impedance-index estimate differed between n = 55 (β = 0.0670; p = 0.0816) and n = 54 (β = 0.0817; p = 0.0131); this finding should be interpreted cautiously.

Table S2. Sensitivity to retaining the participant with invalid adiposity values

Sample	Model	Term	β	95% CI	p	VIF
n = 55	SMM + weight	MUAMC	0.0337	0.0059 to 0.0616	0.0187	5.02
n = 55	Index + weight	MUAMC	0.0670	-0.0087 to 0.1427	0.0816	5.02
n = 54	SMM + weight	MUAMC	0.0355	0.0089 to 0.0621	0.0100	5.16
n = 54	Index + weight	MUAMC	0.0817	0.0180 to 0.1455	0.0131	5.16

Only non-adiposity outcomes are used here; the 0% body-fat values remain excluded from adiposity analyses.

Table S3. Phase-angle models and exploratory sex interaction

Model	Term	β	95% CI	p	VIF
PhA basic	MUAMC	0.0107	0.0060 to 0.0154	<0.001	3.06
PhA basic	Sex	0.4961	0.1866 to 0.8055	0.0023	3.35

PhA basic	Age	-0.0107	-0.0219 to 0.0004	0.0594	1.16
PhA basic	Height	-0.0109	-0.0296 to 0.0078	0.2469	2.63
PhA interaction	MUAMC	0.0042	-0.0009 to 0.0094	0.1075	9.82
PhA interaction	Sex	-1.5816	-3.2409 to 0.0777	0.0613	88.44
PhA interaction	Sex × MUAMC	0.0090	0.0023 to 0.0157	0.0095	130.60
PhA interaction	Age	-0.0103	-0.0209 to 0.0004	0.0581	1.17
PhA interaction	Height	-0.0110	-0.0293 to 0.0072	0.2303	2.63
PhA interaction centred	MUAMC centred	0.0042	−0.0009 to 0.0094	0.1075	9.82
PhA interaction centred	Sex	0.6917	0.3933 to 0.9900	<0.001	4.10
PhA interaction centred	Sex × MUAMC centred	0.0090	0.0023 to 0.0157	0.0095	4.96
PhA interaction centred	Age	−0.0103	−0.0209 to 0.0004	0.0581	1.17
PhA interaction centred	Height	−0.0110	−0.0293 to 0.0072	0.2303	2.63

Sex-by-MUAMC interaction is exploratory; small subgroup sizes and influential observations limit inference.

Table S4. Model diagnostics

Model	RESET p	Quadratic p	BP p	Max Cook D	LOO SD
SMM basic	0.7556	0.4418	0.3225	0.6090	0.0028
SMM + weight	0.0014	0.3024	0.1263	1.9244	0.0019
SMM interaction	0.8288	0.1392	0.3444	0.5887	0.0032
PhA basic	0.0197	0.0276	0.3558	0.1053	0.0003
PhA interaction	0.2278	0.6757	0.3606	0.0827	0.0003
Index basic	0.9347	0.8167	0.2465	0.3767	0.0052
Index + weight	0.1015	0.9591	0.1056	0.8340	0.0051

RESET results suggest possible model misspecification. The leave-one-out (LOO) SD column summarises variation in coefficients from conventional OLS refits; it does not represent HC3-robust LOO p values.

Table S5. Impedance-index regression models

Model	Term	β	95% CI	p	VIF
Index basic	MUAMC	0.1668	0.0905 to 0.2432	<0.001	3.06
Index basic	Sex	1.4721	-2.4569 to 5.4011	0.4552	3.35
Index basic	Age	0.1344	0.0170 to 0.2518	0.0256	1.16
Index basic	Height	0.4360	0.1684 to 0.7036	0.0019	2.63
Index + weight	MUAMC	0.0670	-0.0087 to 0.1427	0.0816	5.02
Index + weight	Sex	5.5806	0.3514 to 10.8098	0.0370	3.75
Index + weight	Age	0.0639	-0.0678 to 0.1956	0.3344	1.22

Index + weight	Height	0.3293	0.0628 to 0.5958	0.0165	2.73
Index + weight	Weight	0.2520	0.0565 to 0.4476	0.0126	2.50

Impedance index was defined as height (cm)^2/R50 (Ω). Results are exploratory and sensitive to model adjustment and sample selection.

Table S6. Bland–Altman regression summary

Group	n	Mean bias (pp)	Slope	Slope p	Residual SD
all	54	1.598	0.1125	0.0167	2.906
men	31	0.939	0.1565	0.0405	2.779
women	23	2.487	0.0260	0.7534	3.077

Differences were defined as BIA minus skinfold-derived percentage body fat. The regression-based conditional limits shown in Figure 2 were constructed using the fitted proportional bias and a constant residual standard deviation. Residual-variance constancy was not independently established and remains a limitation of these conditional limits. No clinically acceptable difference was prespecified.

Table S7. Dominant and non-dominant HGS models

Model	Term	β	95% CI	p	VIF
HGS dominant	Dominant HGS	0.1031	-0.1364 to 0.3426	0.3914	2.35
HGS dominant	Sex	6.3742	1.3492 to 11.3991	0.0140	2.81
HGS dominant	Age	0.0770	-0.0142 to 0.1682	0.0960	1.06
HGS dominant	Height	0.3442	0.2058 to 0.4826	<0.001	2.61
HGS nondom.	Nondom. HGS	0.1245	-0.0623 to 0.3113	0.1866	2.42
HGS nondom.	Sex	6.0554	1.4588 to 10.6520	0.0109	2.96
HGS nondom.	Age	0.0798	-0.0115 to 0.1711	0.0854	1.07
HGS nondom.	Height	0.3433	0.2026 to 0.4841	<0.001	2.54

One right-hand measurement followed by one left-hand measurement; no familiarization trial. HGS reported in kgf (conventionally kg).

Table S8. Rank correlations and sample sensitivity

Sample	Predictor	Outcome	n	Spearman ρ	p
n = 55	MUAMC	BIA-derived skeletal muscle mass (kg)	55	0.9129	<0.001
n = 55	MUAMC	Phase angle (°)	55	0.8063	<0.001
n = 55	MUAMC	Impedance index (cm^2/Ω)	55	0.8946	<0.001
n = 55	Dominant HGS	BIA-derived skeletal muscle mass (kg)	54	0.7695	<0.001
n = 55	Nondom. HGS	BIA-derived	54	0.7717	<0.001

		skeletal muscle mass (kg)			
n = 55	Skinfold-derived body fat (%)	BIA-derived body fat (%)	54	0.9125	<0.001
n = 54	MUAMC	BIA-derived skeletal muscle mass (kg)	54	0.9094	<0.001
n = 54	MUAMC	Phase angle (°)	54	0.8125	<0.001
n = 54	MUAMC	Impedance index (cm^2/Ω)	54	0.9028	<0.001
n = 54	Dominant HGS	BIA-derived skeletal muscle mass (kg)	53	0.7689	<0.001
n = 54	Nondom. HGS	BIA-derived skeletal muscle mass (kg)	53	0.7704	<0.001
n = 54	Skinfold-derived body fat (%)	BIA-derived body fat (%)	54	0.9125	<0.001

Unadjusted Spearman correlations; pooled associations should not be interpreted as independent clinical validation.

Table S9. Within-sex Spearman correlations

Pair	Men: n, ρ (95% CI)	Women: n, ρ (95% CI)	Bootstrap
MUAMC–SMM	32; 0.748 (0.518 to 0.881)	23; 0.673 (0.306 to 0.890)	1,800; seed 42
MUAMC–PhA	32; 0.612 (0.288 to 0.816)	23; 0.164 (−0.318 to 0.561)	1,800; seed 42
Dominant HGS–SMM	32; 0.376 (0.013 to 0.670)	22; 0.566 (0.206 to 0.791)	1,800; seed 42

Paired percentile bootstrap 95% confidence intervals; 1,800 resamples; random seed 42.

Table S10. Within-sex, age- and height-adjusted MUAMC models, with additional weight-adjusted SMM models

Sex	Outcome	n	β per mm (95% HC3 CI)	HC3 p
Men	SMM	32	0.1086 (0.0481 to 0.1690)	0.0010
Women	SMM	23	0.0987 (0.0464 to 0.1511)	0.0009
Men	PhA	32	0.0140 (0.0082 to 0.0199)	<0.001
Women	PhA	23	0.0033 (−0.0026 to 0.0092)	0.2535
Men	SMM + weight	32	0.0360 (0.0036 to 0.0684)	0.0307
Women	SMM + weight	23	0.0281 (−0.0021 to 0.0595)	0.0657

Centred interaction model: additional parameterization reported in Table S3

MUAMC was centred on the pooled mean (253.175 mm). The sex × centred-MUAMC interaction was β = 0.00898 degrees/mm (95% HC3 CI 0.0023–0.0157; p = 0.0095). VIFs: sex 4.10, centred MUAMC 9.82, interaction 4.96, age 1.17, height 2.63. Centring changes parameterization, not the interaction estimate or its inferential p value.

Influence sensitivity: weight-adjusted SMM model

Sample	β MUAMC (kg/mm)	95% HC3 CI	p
All n = 55	0.0337	0.0059 to 0.0616	0.0187
Without zero-fat participant, n = 54	0.0355	0.0089 to 0.0621	0.0100
Without Cook D = 1.924 observation, n = 54	0.0436	0.0241 to 0.0632	<0.001

The participant with a device-reported 0% body-fat estimate and the observation with maximum Cook's distance were different individuals. The underlying cause of the 0% BIA estimate could not be determined retrospectively; internal phase-angle consistency does not independently validate device-derived SMM.

Exploratory IBS symptom analyses

The mean daily number of diarrhoeal stools was not significantly associated with phase angle (Spearman ρ = −0.108; p = 0.431; n = 55). This exploratory analysis is not interpreted as a primary clinical finding. IBS-SSS is presented descriptively in the main manuscript; the unpublished exploratory IBS-SSS regression is not reported.

Reproducibility

- Analyses were implemented in Python using NumPy, pandas, SciPy, statsmodels and openpyxl; software requirements and execution instructions are documented in the accompanying code archive.
- The analytical sample included 55 participants for valid non-adiposity variables, 54 for body-fat comparisons and 54 with available dominant-hand HGS.
- The invalid zero body-fat outputs were excluded from adiposity analyses only.
- Individual participant-level data are not publicly distributed; access is subject to the conditions described in the manuscript's Data Availability Statement.
